# Supplementary material for: The interactome of intact mitochondria by cross-linking mass spectrometry provides evidence for coexisting respiratory supercomplexes
Source: Mol Cell Proteomics. 2017 Dec 8;17(2):216–32. doi: 10.1074/mcp.RA117.000470 (PMC5795388; doi:10.1074/mcp.RA117.000470)
Supplement: Supplemental Data [file supp_RA117.000470_133922_0_supp_23978_fzffwf.zip › spectra_annotation/mito_DR_spectra_annotation/157-1-3-1-7-1.pdf]

KAVTPAPPMK
KYTEQITNEK

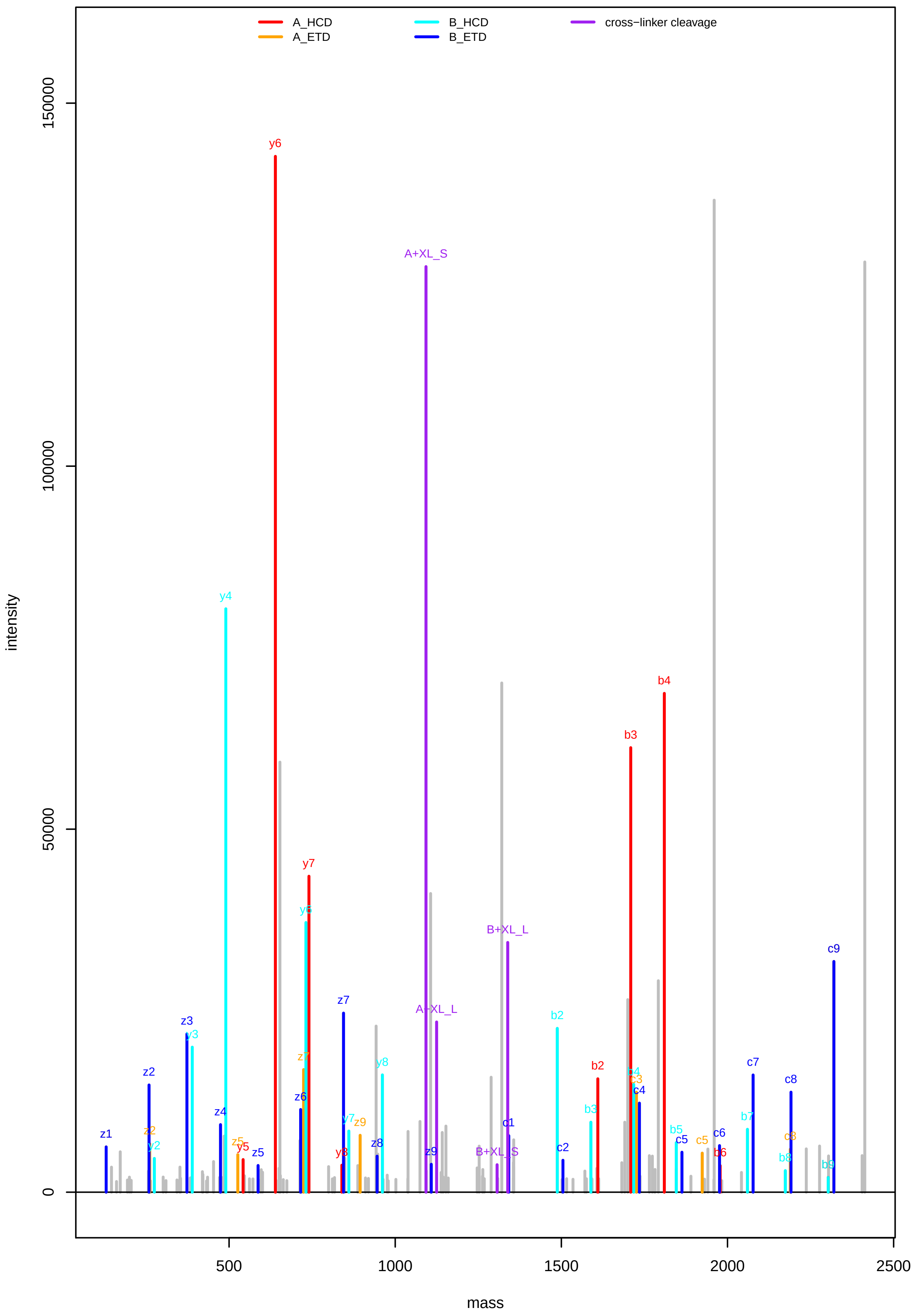

KAVTPAPPMK+XL_S

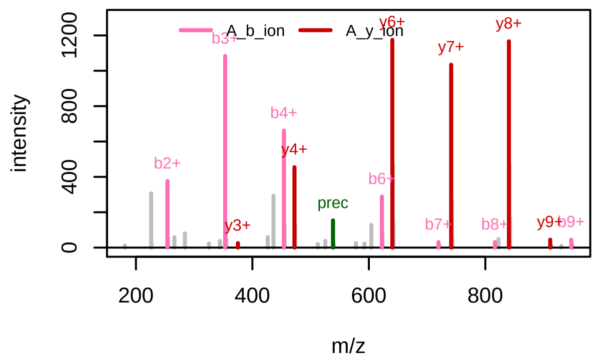

KAVTPAPPMK+XL_L

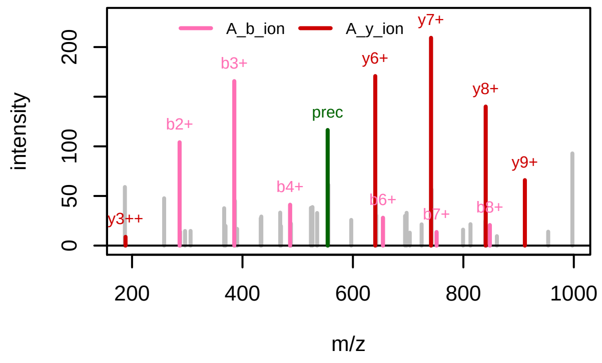

KYTEQITNEK+XL_S

KYTEQITNEK+XL_L
